# Annual Commencement of the Dental Department of the University of Maryland

**Published:** 1886-03

**Authors:** 


### Editorial, Etc.

ANNUAL COMMENCEMENT OF THE DENTAL DEPARTMENT OF THE UNIVERSITY OF MARYLAND.—The Annual Commencement of the University of Maryland, Dental Department, in connection with the seventy-ninth Annual Session of the University School of Medicine, was held at the Academy of Music, Baltimore, on Wednesday, March 17th, 1886. The reading of the mandamus and the announcement of the Graduates by Professor Ferdinand J. S. Gorgas, Dean. The degree of D. D. S., was conferred by Hon. S. Teackle Wallis, L. L. D., Provost of the University, upon the following gentlemen, all of whom had attended two full sessions of five months each in separate years:

| | |
|---|---|
| Amend, Emil | Germany |
| Baden, Frank A. | Maryland |
| Basehore, Horace E. | Pennsylvania |
| Brugeille, Emile | France |
| Bookhart, Thomas W. | South Carolina |
| Bruce, William W. | West Virginia |
| Campbell, Oscar J. | Virginia |
| Chafee, Augustus H. | South Carolina |
| Diehl, John S. | Pennsylvania |
| Emerson, Joseph G. | Brazil, S. Am |
| Furman, Charles Luff | New York |

| | |
|---|---|
| Gasque, Elly A. | South Carolina |
| Greenawalt, D. D. S., A. H. | Pennsylvania |
| Hartwig, Charles W. | Maryland |
| Hoffman, John H. | Virginia |
| Huggins, G. Allen | South Carolina |
| Lowell, William H. | Pennsylvania |
| Lumsden, Frank H. | Maryland |
| Macgill, Jr., Lloyd T. | Maryland |
| Pleasants, Wilfred A. | Virginia |
| Proctor, Jr., W. Eppes | Virginia |
| Purnell, Ralph C. | Maryland |
| Riley, James M. | North Carolina |
| Shields, Lewis N. | Texas |
| Sims, Benjamin F. | South Carolina |
| Slocum, Frank E. | New York |
| Wall, Joseph A. | Pennsylvania |

The University Prize, Gold Medal, for the highest number of votes at the final examination, was awarded to William E. Proctor, Jr., of Virginia. Honorable mention was awarded to Wilfred A. Pleasants, of Virginia.

The Address to the Graduates was delivered by Col. William Allen, President of McDonough Institute.

The number of Matriculates for the Session of 1885-86, was *ninety-one*.

Although the number of Matriculates for the Session was larger than ever before, the number of graduates is smaller, on account of a strict compliance with the *two session rule*, as a requisite for graduation, no less than *twenty-two* having been refused, who desired to graduate in one session, on 5 years or more of practice.

The annual meeting of the Alumni Association of the Dental Department, University of Maryland, was held at the Howard House, Thursday evening, March 18th. Dr. Charles L. Steel, of Virginia, presided. Prof. F. J. S. Gorgas addressed the meeting. Members of class of 1886 and a number of the graduates of other colleges were elected members. The following officers were elected for ensuing year: President, Dr. R. D. Dodson, Pennsylvania; vice-president, Dr. J. Fournien, N. Y.; secretary, Dr. J. S. Kloeber, of Virginia; treasurer, Dr.

J. H. Davis, of Maryland. A banquet followed. Many speeches were made.

The contest for prizes by members of the graduating class of the Dental Department of the University of Maryland took place Saturday, March 13th, 1886. The judges consisted of a number of prominent dental practitioners from Maine, Virginia, Pennsylvania, District of Columbia, North Carolina, Maryland, New Jersey, West Virginia and other States, who came to Baltimore for the purpose. The prizes were awarded for practical work in the infirmary and laboratory, and are as follows:

1. S. S. White prize, dental engine, for best full upper set of gum teeth on metal base, to W. H. Lowell, of Pennsylvania; honorable mention, J. H. Hoffman.

2. Snowden & Cowman prize, set of C. A. Harris' forceps, for best partial set of five or more teeth on metal base, to W. H. Lowell; honorable mention, L. N. Shields.

3. S. S. White prize, set of Varney's pluggers, for best two fillings of cohesive and non-cohesive gold, to E. Brugeille; honorable mention, L. N. Shields.

4. Dr. T. L. Wood prize, gold medal, for best contour gold filling, to W. H. Lowell; honorable mention, E. A. Gasque.

5. Dr. N. T. Shield prize, gold medal, for best filling of non-cohesive and cohesive gold combined, to W. A. Pleasants; honorable mention, W. E. Proctor, Jr.

6. Dr. Jas. H. Harris prize, gold medal, for best filling of Abbey's non-cohesive gold, to J. G. Emerson; honorable mention, C. L. Furman.

7. Dr. F. J. S. Gorgas prize, second edition of Gorgas' Dental Medicine, for best combination set of metal and either vulcanite or celluloid, with soldered rim, to W. W. Bruce; honorable mention, H. E. Basehore.

8. Dr. B. H. Catching prize, one year's subscription to *Southern Dental Journal*, best examination on dental materia medica and therapeutics, to W. E. Proctor, Jr.

9. Dr. J. Uhler prize, gold medal, best set of teeth of continuous gum, to E. Brugeille; honorable mention, John H. Hoffman.

10. Dr. C. L. Steel prize, gold medal, best specimen tooth filling, W. H. Lowell.

